# Effect of the Severe Plastic Deformation on the Corrosion Resistance of a Tantalum–Tungsten Alloy

**DOI:** 10.3390/ma15217806

**Published:** 2022-11-05

**Authors:** Guoqiang Ma, Man Zhao, Song Xiang, Wanquan Zhu, Guilin Wu, Xinping Mao

**Affiliations:** 1 Beijing Advanced Innovation Center for Materials Genome Engineering, University of Science and Technology Beijing, Beijing 100083, China; 2 College of Materials Science and Engineering, Chongqing University, Chongqing 400044, China; 3 College of Materials and Metallurgy, Guizhou University, Guiyang 550025, China; 4 Yangjiang Branch, Guangdong Laboratory for Materials Science and Technology (Yangjiang Advanced Alloys Laboratory), Yangjiang 529500, China

**Keywords:** tantalum alloy, severe plastic deformation, grain subdivision, corrosion, crystallographic orientation

## Abstract

Tantalum and its alloys are regarded as equipment construction materials for processing aggressive acidic media due to their excellent properties. In this study, the influence of severe rolling (90%) on the dissolution rate of a cold-rolled Ta-4%W sheet in different directions was investigated during immersion testing and the corresponding mechanism was discussed. The results show that the dissolution rate of the cold-rolled sample is significantly lower than that of the undeformed sample. The corrosion resistance followed the sequence of “initial” < “90%-ND” < “90%-RD” < “90%-TD”, while the strength is in positive correlation with the corrosion resistance. Severe rolling promotes grain subdivision accompanied by long geometrically necessary boundaries and short incidental dislocation boundaries on two scales in the cold-rolled sample. The volume elements enclosed by geometrically necessary boundaries form preferential crystallographic orientations. Such preferential crystallographic orientations can greatly weaken the electrochemical process caused by adjacent volume elements, resulting in greatly reduced corrosion rates in the severely deformed sample. The unexpected finding provides a new idea for tailoring the structures of tantalum alloys to improve both their strength and corrosion resistance.

## 1. Introduction

Tantalum (Ta) is a typical rare transition metal. Ta and its alloys have received widespread attention in many fields such as nuclear engineering, the chemical industry, biomaterials, high temperature applications and the electronics industry. This is mainly because Ta and its alloys have excellent properties including high melting points, high corrosion resistance, good biocompatibility, mechanical and electrical properties [1,2,3,4,5,6]. Due to the excellent corrosion resistance, Ta alloy mill products, especially the binary Ta alloys containing tungsten (W), are used to manufacture processing equipment meant for harsh chemical environments, for example, bayonet heaters, tank liners, heat exchangers and various other components for sulfuric acid production [7]. While many previous studies have mainly focused on the corrosion behaviors of Ta and its alloys in hot, highly corrosive environments [8,9,10,11], limited attention has been paid to the relationship between thermomechanical processing and corrosion resistance.

Regardless of the final form of Ta alloy mill products, thermomechanical processing is an essential process. During the thermomechanical processing, the alloy undergoes different forms and/or levels of plastic deformation. Strain path and strain degree, etc., affect the microstructure and final properties of the alloy. According to earlier research results, the corrosion rate increases when metals and alloys undergo plastic deformation [12,13,14]. The theoretical basis for such results is that dislocations and other defects are generated during the deformation. These defects usually increase the active sites on the metal surface [14]. Plastic deformation increases the rate of anode dissolution but has little effect on the cathode process [13]. For example, Luo et al. [15] investigated a duplex stainless steel in a saturated Ca(OH)_2_ solution containing 3.5 wt.% NaCl and noted that plastic deformation accelerated the corrosion rate due to the enhancement of metastable pitting susceptibility. Stefec et al. [16] adopted quantitative metallography to statistically analyze the corrosion pits and found the total area and number of pits increased with increasing deformation.

In recent years, a series of breakthroughs have been realized in tailoring nanostructured grains to achieve both high strength and good ductility by severe plastic deformation [17]. However, severe plastic deformation also introduces large proportions of metastable microstructures, grain boundaries and textures, which leads to a more complex corrosion behavior compared to conventional polycrystalline metals and alloys [18]. Wang et al. [19,20] obtained nanocrystalline ingot iron after 93% rolling reduction from conventional polycrystalline and found that the corrosion resistance of the obtained samples was greatly improved in both 1 M HCl and 0.05 M H_2_SO_4_ + 0.25 M Na_2_SO_4_ solutions. Lv et al. [21] found that pure iron had similar textures after a rolling reduction of 95.8% at room temperature and in liquid nitrogen conditions, but the grain refinement between them was different. Compared to the original annealed sample, the corrosion resistances of rolled samples were both improved, but the corrosion resistance of the sample rolled at room temperature was better than that of the sample rolled in liquid nitrogen.

The above results show that the presence of metastable microstructures, dislocations, and dislocation boundaries increase the corrosion rate, while the formations of textures and changes in the second phase may inhibit the corrosion rate during deformation. Recently, the potential to improve corrosion resistance by severe plastic deformation has been demonstrated in several separate studies for different metals and alloys. However, the interpretive reasons are different for such improvements [22,23,24,25]. The question still remains therefore how the change in corrosion resistance relates to the severe deformation structure for metals and alloys. In our previous studies on the Ta-4%W alloy [26,27,28,29,30], we have reported the microstructural evolution after cold rolling and the corrosion behavior on rolling plane in sulfuric acid. However, the effects of macroscopic orientation and crystallographic texture in different directions on the surface dissolution of cold-rolled sheets are still unknown and they are the key components of the present research.

In this study, corrosion rates in different macroscopic orientations of the cold-rolled sample were tested, and the corrosion morphology and microstructure were characterized. The study focuses on three aspects: (1) a corrosion degree comparison in different macroscopic orientations after severe rolling, (2) the microstructural source of the texture and (3) the relationships between texture and corrosion behavior. Our paper represents the first investigation to establish grain subdivision, dislocation substructure and texture evolution, as well as to establish a direct relationship to corrosion resistance and mechanical properties.

## 2. Materials and Methods

Ta-4%W ingots were obtained from electron beam melting under high-vacuum conditions. Ingots are forged and annealed in preparation for starting material, which has been described in our previous work [28,30]. The starting plates have a thickness of 10 mm, and then they are rolled at room temperature to a reduction in thickness of 90% (corresponding to a von Mises strain of 2.7). The rolled sheet has a thickness of 1 mm after rolling. In order to reveal the corrosion behaviors on different sections of the rolled sheet, i.e., sections perpendicular to the rolling direction (RD), the transverse direction (TD) and the normal direction (ND), the samples for immersion corrosion were cut from the rolled sheet with a size of 10 mm in length, 1 mm in width, and 0.3 mm in thickness. To obtain precision results, 9 samples were prepared for each tested section.

**Figure 1:**
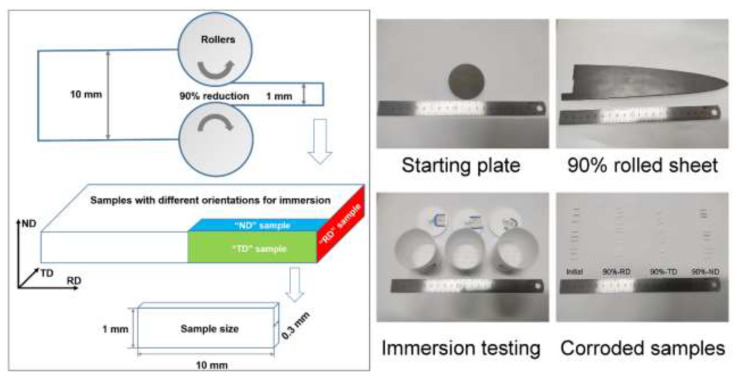
Schematic illustration and experimental pictures of samples for immersion testing: the “initial” sample was cut from the 10 mm-thick plates before cold rolling. The observed surfaces of the “90%-RD”, “90%-TD” and “90%-ND” samples were perpendicular to the rolling direction, transverse direction and normal direction, respectively, of the 90% cold-rolled plate.

All the samples were grounded with final 5000 grit sandpaper, cleaned with alcohol and dried with a hair dryer before the corrosion tests. Then, the samples were immersed in a mixture solution of hydrofluoric acid, sulfuric acid and deionized water (1:50:49 by mass) from 7 days to 30 days. To explore the effect of severe plastic deformation on the corrosion rate of Ta-4%W, the initial undeformed sample was also immersed in the test solution and cut to the same size as the deformed sample to avoid possible size effects. The schematic illustration and experimental pictures of the samples’ preparation for immersion testing is shown in Figure 1. The samples were weighed with a high precision electronic scale both before and after immersion testing in order to record the mass loss of the alloy, and then the total weight changes for each tested section were converted into the corrosion rate, which is calculated according to the following equation:

X (mm/a) = 87600×/(W/DAT)(1)

where W is the weight loss in grams, D is the sample density taken to be 16.6 g⋅cm^−3^, A is the area of the sample in cm^2^, and T is the time of exposure of the Ta-4%W sample in hours. After immersion, the micro-hardness of each test surfaces was also measured using a load of 500 g and a dwell time of 10 s. The corrosion morphologies of each sample immersed in the test solution after 30 days were characterized by a ZEISS Auriga scanning electron microscope (SEM) attached with an electron backscatter diffraction (EBSD) detector. Transmission electron microscope (TEM) foils were observed in a JOEL JEM 2100 TEM equipped with an online Kikuchi-line analysis system for crystallographic orientation determination [31].

## 3. Results and Discussion

**Figure 2:**
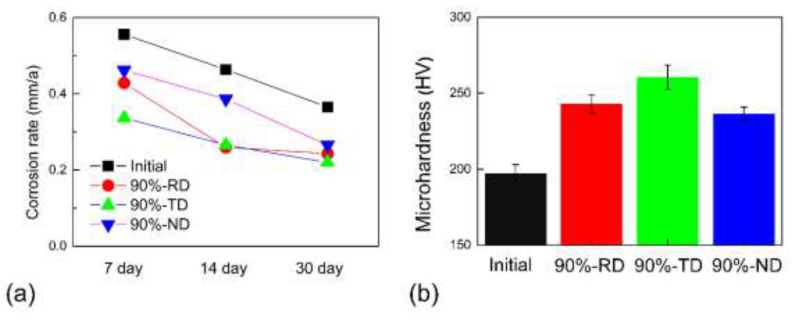
The corrosion rates of Ta-4%W in a 50 wt.% H_2_SO_4_ solution containing 1 wt.% fluoride ions (**a**) and the micro-hardness of the corresponding test surfaces (**b**).

Figure 2 shows the corrosion rates of samples in the test solution from 7 days to 30 days and the micro-hardness of the corresponding test surfaces. As shown in Figure 2a, the corrosion rates of all the samples decrease with increasing corrosion time. This may be due to corrosion products gradually accumulating and covering the surfaces of the tested samples. Ta alloys show excellent passivation tendency due to a protective passive stable Ta_2_O_5_ oxide film. However, when Ta alloys are immersed in a mixture of hydrofluoric and sulfuric acid, several nanometers of Ta_2_O_5_ on the sample surface will dissolve and a corrosion product of fluoride complex (H_2_TaF_7_) will form. Such corrosion products act as a barrier to further corrosion [11], thus slowing down the corrosion process. Another surprising result is that the corrosion rates of the cold-rolled sample are significantly smaller than those of the initial sample regardless of the macroscopic orientations of the test samples. Among the cold-rolled samples in different orientations, the corrosion rate of the 90%-ND sample is higher than those of the 90%-RD sample and the 90%-TD sample. The micro-hardness results show that the 90%-TD sample has a maximum value of 261 HV, while the initial sample has a minimum value of 197 HV (Figure 2b). Generally, the corrosion resistance is in an inverse correlation with the strength. However, present results seem to support the conclusion that the harder the surface, the more resistant it is to corrosion. Similar results were also seen in BN-304SS by severe rolling technology [32].

**Figure 3:**
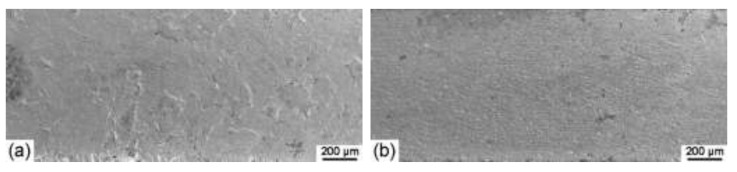

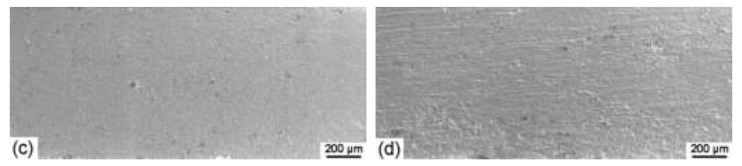
SEM images of the Ta-4%W corrosion samples after 30 days of immersion testing: (**a**) “initial” sample, (**b**) “90%-RD” sample, (**c**) “90%-TD” sample, and (**d**) “90%-ND” sample.

Figure 3 shows SEM images of the morphologies for the Ta-4%W samples after the immersion test. The corroded surface is uneven between the adjacent grains, which means the shape of the grains is faintly visible for the initial sample (Figure 3a). After 90% cold rolling, the corroded surfaces of the samples with three macroscopic orientations become quite uniform. Based on the comparison of the corrosion morphology for each of the samples, the “90%-TD” sample can be assumed as relatively less corroded, followed by the “90%-RD” sample, then the “90%-ND” sample, while the initial sample can be assumed as more corroded.

**Figure 4:**
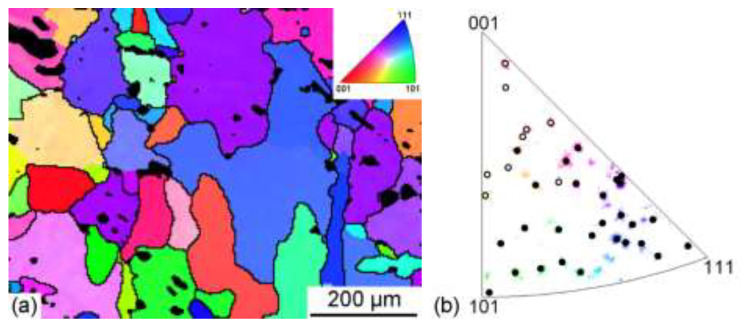
EBSD orientation maps (**a**) and IPF (**b**) of grains detected in the initial sample after 30 days of immersion testing. Note that the black regions in (**a**) are the preferentially corroded sites which cannot be resolved by EBSD. The grains without corroded sites are marked as hollow circles while the grains with corroded sites are marked as solid black circles in (**b**).

To further understand the difference in corrosion resistance, the corroded surfaces after 30 days of immersion testing were also characterized using EBSD. Due to a high density of 16.6 g/cm^3^ for the present alloy, a high absorption ratio will happen when the electron beam falls into the pit areas. Thus, the surface quality of Ta alloys has a significant impact on the detection of EBSD signals, and the corroded sites or pit areas on corroded surfaces formed in immersion testing will not be resolved by EBSD [33]. Figure 4 shows the EBSD orientation maps and inverse pole figure (IPF) of grains detected in the initial sample. As shown in the orientation maps (Figure 4a), “blue” and “green” grains tend to have more severely corroded sites or pit areas (indicated by the black color), while “red” grains have fewer pit areas. The orientations of all the grains detected are shown in Figure 4b. It can be concluded that the {100} grains exhibit a significantly lower corrosion degree compared with {110} and {111} grains, which can also be supported by previous studies for Ta [30,34] and other body-centered cubic (bcc) metals such as Nb [35] and pure Fe [21,36].

**Figure 5:**
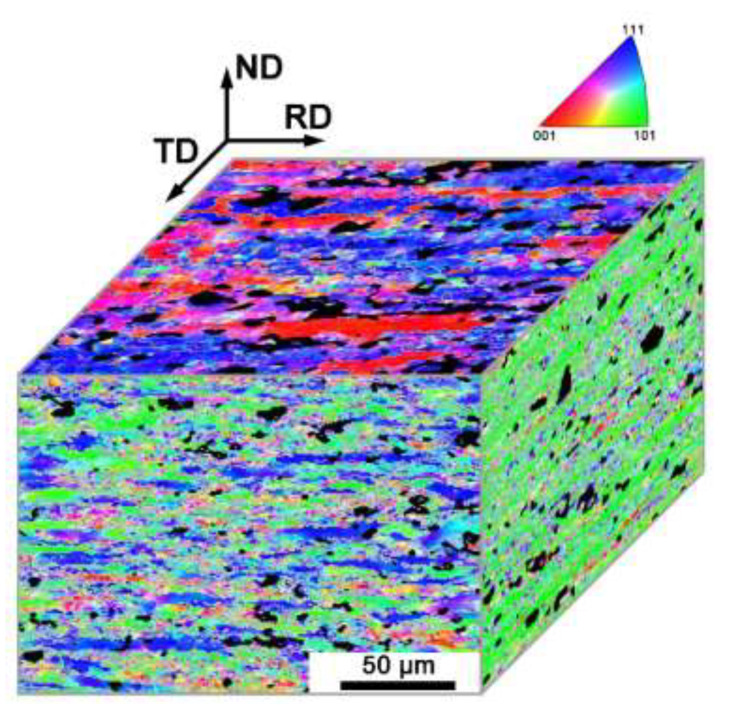
EBSD observation of preferentially corroded sites in the 90% cold-rolled samples after 30 days of immersion testing.

Figure 5 shows EBSD maps of the cold-rolled sample on corroded surfaces. Grain subdivision occurs in three directions and there are many dislocation substructures formed on the deformed sample. It is important that preferential corrosion still exists as the initial sample. The preferentially corroded sites, i.e., the black regions which cannot be detected by EBSD, are primarily located in the {111} grains.

**Figure 6:**
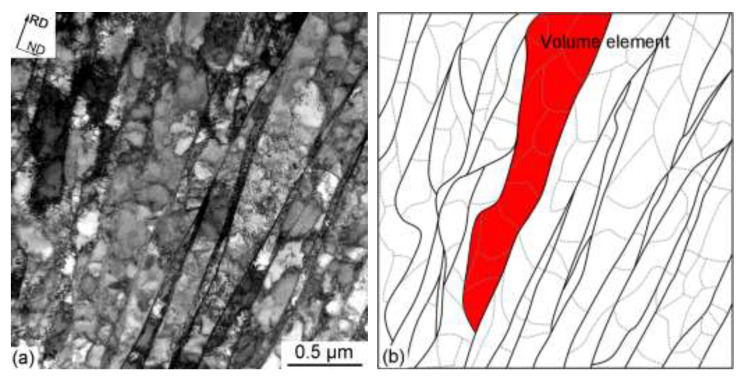
The microstructure of the 90% cold-rolled sample in the longitudinal plane: (**a**) TEM images of the lamellar boundary structure, (**b**) a sketch of the GNBs (solid black lines) and the IDBs (dashed grey lines) in (**a**).

Due to the resolution limit of the EBSD technique, finer-scale characterization of the deformation microstructure was studied using TEM (see Figure 6). Severe rolling promotes grain subdivision containing two types of dislocation boundaries. One is the long and extended planar boundaries with a larger scale, and the other is the highly curved morphology dislocation boundaries with a smaller scale (Figure 6a). The long and extended planar dislocation boundaries are known as geometrically necessary boundaries (GNBs), while the highly curved morphology dislocation boundaries are known as incidental dislocation boundaries (IDBs) [37]. The GNB misorientation angle can reach quite high values (average value of 11.9°) to accommodate lattice rotations and can lead to a strong texture evolution (preferential crystallographic orientations) [28]. The IDB misorientation angles (average value of 3.1°) are much smaller than those of GNB and cannot causes significant crystallographic orientation change across the boundary. A sketch of GNBs and IDBs has been shown in Figure 6b.

Regarding the effect of plastic deformation on the corrosion behavior, a considerable investigation was carried out in a previous study but conflicting conclusions were reached. For example, experimental results by Stefec, Haraszit and Maric et al. [12,16,38] show that plastic deformation reduces the corrosion resistance of austenitic stainless steels as the strain increases (mostly from low to medium strain). Wang et al. [39] found that plastic deformation reduces the corrosion resistance of CrCoFeMnNi high entropy alloys even when the strain reaches 80%. However, experimental results by Lv and Wang et al. [32,40] show that plastic deformation improves the corrosion resistance of austenitic stainless steels when the strain is high enough. The phenomenon of improved corrosion resistance due to plastic deformation is also found in BCC iron [19,20,21]. There are few controversial views on the explanation of plastic deformation reducing corrosion resistance, i.e., numerous dislocations and other crystallographic defects formed after plastic deformation promoting the electrochemical dynamics increase and dissolution rate. However, to the best of our knowledge, no unified view has been reached on the explanation of plastic deformation enhancing corrosion resistance. These mutually contradictory conclusions [19,20,21,22,24,25,32,40] mainly relate to (1) the conventional microstructure parameters such as dislocation density, grain size, twin boundaries, high angle boundaries, second phase, (2) the passivation film properties, and (3) even the valence electron configuration at an atomic scale.

It is worth noting that grain subdivision based on volume scales of dislocation boundaries classification and low energy dislocation structures has been observed in many metals and alloys. At certain strain levels (mostly at a von Mises strain of 0.8), the typical microstructural features of S-bands will form due to the emergence of new slip systems [41,42]. The S-band can also lead to the deformation texture being formed and the texture will continue to strengthen with increasing strain. A more efficient source of high-angle boundaries can be found if the overall texture evolution is considered, rather than just the evolution of individual directions. Therefore, grain subdivision, accompanied by a strong texture evolution, can lead to a significant increase in the fraction of high-angle boundaries during deformation [41]. Volume elements are defined as the regions enclosed by high-angle boundaries in TEM [31]. Since most high-angle boundaries belong to the GNBs, the volume elements enclosed by GNBs can be thus assumed to have the same electrochemical properties since they have the same crystallographic orientation and operate the same glide systems. One of the volume elements is illustrated by the color red in Figure 6b.

**Figure 7:**
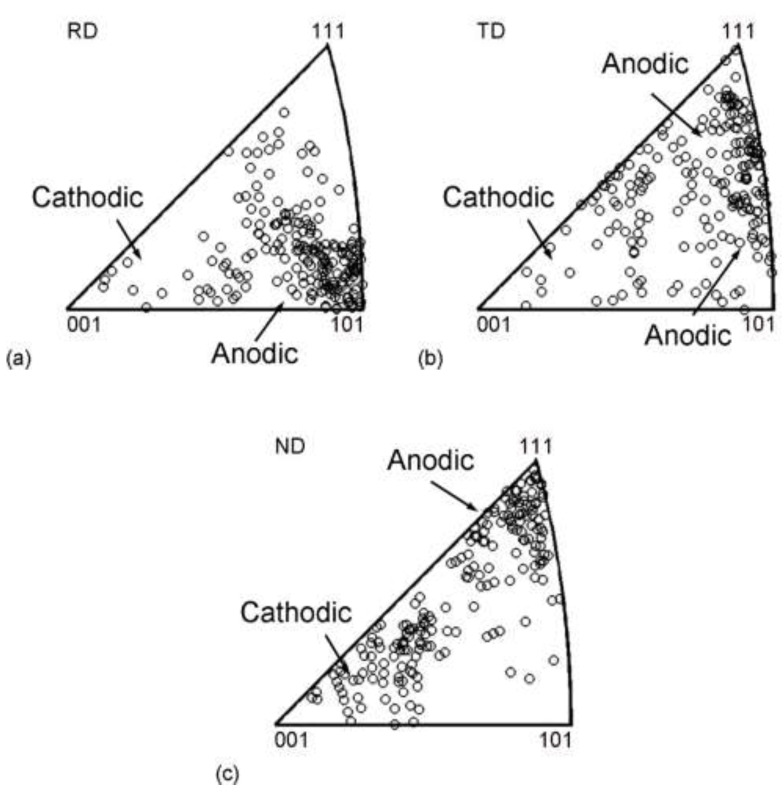
Inverse pole figure showing the (**a**) rolling direction, (**b**) transverse direction, and (**c**) normal directions of the subdivided grains in the 90% cold-rolled samples mapped by TEM.

With the help of an online Kikuchi-line analysis system equipped on a JOEL JEM 2100 TEM [31], the crystallographic orientations of individual volume elements were measured and mapped to inverse pole figure (Figure 7). The crystallographic orientation with RD tends to be <110> in the unit triangle. The crystallographic orientation with TD ranges from <110> to <111> along the edge of the unit triangle. The crystallographic orientation with ND tends to be <111> primarily and tends to be <100> minorly. It is known that the <111> grains have the maximum Taylor factor value, while the <110> grains follow, and the <100> grains have the minimum Taylor factor value. Taylor factor is a parameter that reflects the difficulty of plastic deformation, representing the numbers of activated slip systems during deformation. Thus, the difference in micro-hardness between the macroscopic tests’ surfaces is mainly due to different grain orientation distribution [13].

Compared to the uniform distribution of orientation in the initial undeformed sample (Figure 4b), the cold-rolled samples show significant preferential crystallographic orientations regardless of the sample direction on the rolled sheet. Previous studies have shown that the cathodic process preferred to occur in the <100> orientation volume element, while the anodic process mainly occurs in the <110> and <111> orientation volume element [30,35]. Such preferential crystallographic orientations in cold-rolled samples can greatly weaken the electrochemical process caused by the adjacent volume elements, resulting in greatly reduced dissolution rates for the Ta-4%W alloy. Among the cold-rolled samples, the “90%-ND” sample exhibited a worse corrosion resistance than that of the “90%-RD” and “90%-TD” samples. The possible reason for this result is the fact that the “90%-ND” sample has more <100> volume element than the “90%-RD” and “90%-TD” samples.

## 4. Conclusions

The Ta-4%W alloy was cold rolled to achieve a reduction of 90% in sample thickness. The effect of severe plastic deformation on the corrosion behavior of the Ta-4%W alloy in a mixed solution of sulfuric acid and hydrofluoric acid was investigated. The results show that 90% cold rolling can significantly reduce the dissolution rate of the Ta-4%W alloy regardless of the macroscopic orientation of the cold-rolled samples. The corrosion resistance followed the sequence of “initial” < “90%-ND” < “90%-RD” < “90%-TD”. During plastic deformation, two types of dislocation boundaries were formed. One type was the long and extended planar boundaries (GNBs) which subdivide the grains at a larger scale. The other type was the highly curved morphology dislocation boundaries (IDBs) formed between GNBs. Preferential crystallographic orientations were formed through volume elements enclosed by GNBs. The electrochemical process is different due to the difference in crystallographic orientations between the adjacent volume elements. However, the preferential crystallographic orientation might weaken this difference and thus greatly reduce the corrosion rate of the Ta-4%W alloy.
